# Bioactive-Supplemented Infant Formulas and Early Gut-Immune-Endocrine Development: A Narrative Review

**DOI:** 10.3390/ijms27104613

**Published:** 2026-05-21

**Authors:** Salvatore Scirè Calabrisotto, Roberta Leonardi, Marco Guercio, Martina Barbato, Caterina Carpinato, Carmine Mattia, Nunzia Decembrino, Grazia Maria Palano, Martino Ruggieri, Pasqua Betta

**Affiliations:** 1 Postgraduate Residency Program in Pediatrics, Department of Clinical and Experimental Medicine, University of Catania, 95123 Catania, Italy; s.scire.doc@gmail.com (S.S.C.); marco.guercio1@gmail.com (M.G.); martinabarbato98@gmail.com (M.B.); 2 Neonatal Intensive Care Unit (NICU), A.O.U. Policlinico “G. Rodolico-San Marco”, P.O. “G. Rodolico”-University of Catania, 95123 Catania, Italy; g.mannino@tiscali.it (C.C.); lorettamattia@hotmail.com (C.M.); ninnidecembrino@gmail.com (N.D.); graziamaria.palano@gmail.com (G.M.P.); mlbetta@yahoo.it (P.B.); 3 Unit of Pediatric Clinic, A.O.U. Policlinico “G. Rodolico-San Marco”, P.O. “G. Rodolico”-University of Catania, 95123 Catania, Italy

**Keywords:** infant nutrition, bioactive-supplemented infant formula, gut microbiota, human milk oligosaccharides, prebiotics, probiotics, postbiotics, immune development, infant growth, gut–brain axis

## Abstract

Nutrition in the early years of life plays a fundamental role in newborn growth, immune maturation, metabolic regulation, endocrine signaling, and neurological development, specifically through its interaction with the developing gut microbiota. Breast milk is the biological gold standard for infant nutrition; however, when breastfeeding is not possible, the development of formulations supplemented with bioactive substances can improve functional outcomes in comparison to standard milk formula. This narrative review discusses current evidence on formulas enriched with prebiotics, probiotics, postbiotics, synbiotics, human milk oligosaccharides, and other bioactive molecules. The review focuses on gut microbiota modulation, gastrointestinal function, growth and nutritional adequacy, immune development, infection-related outcomes, safety and tolerability, endocrine signaling, intestinal stem-cell regulation, obesity-related metabolic pathways, and emerging gut–brain axis interactions. Overall, available data indicate that bioactive-supplemented formulas are generally safe, well tolerated, and able to support normal growth, including in selected infants with specific clinical conditions. The most consistent effects are observed in the gastrointestinal tract, where supplementation promotes a more bifidogenic microbial profile, improves stool characteristics, supports intestinal barrier function, and influences microbial metabolic activity. By contrast, evidence regarding systemic immune effects, endocrine modulation, obesity prevention, and neurodevelopmental outcomes remains promising but heterogeneous and is still largely derived from preliminary human studies and experimental models. Therefore, these formulas may be considered a useful option when breastfeeding is not feasible, provided that their use is clinically appropriate and evidence based. Further studies are needed to clarify their long-term functional and clinical implications.

## 1. Introduction

“You are what you eat”, a well-known and straightforward phrase that effectively reflects the importance of nutrition for our health. Since 2008, starting from the Lancet Series on Maternal and Child Undernutrition, scientific literature has begun stressing the importance of the first 1000 days of life as a critical window for adult well-being [1]. In fact, it is reported a major impact of nutrition during the first 1000 days of life, namely on non-communicable diseases [2], neurocognitive development [3], allergies [4], immune disorders, even if non-consistent data are available [5,6], and also in adult economic and social status [7]. Breastfeeding represents the cornerstone of infant nutrition. Scientific literature recommends exclusive breastfeeding for at least the first six months, supporting also the extension up to the first two years [8,9], due to the known positive human milk impact on growth rate, neurocognitive outcomes, and in reducing infectious morbidity [3,10,11].

Human milk, beyond a balanced macro and micronutrient composition, contains a wide array of bioactive components, namely secretory immunoglobulins, immune cells, cytokines, growth factors, hormones, and a variety of human milk oligosaccharides (HMOs), which, altogether, influence positively immune and metabolic maturation [12,13,14,15].

Unfortunately, breastfeeding happens to be not feasible or not indicated in some mother or baby-related issues. Therefore, infant formula, enriched with bioactive molecules, such as HMOS, galactooligosaccharide (GOS), fructooligosaccharide (FOS), probiotics, prebiotics, or postbiotics might be an appealing alternative, being capable of reproducing breastfeeding positive effects and positively modulating gut resident microbiota.

In recent years, HMOs’ active biological components received increased attention, due to their relevance in many biochemical pathways. HMOs represent the third most present solid fraction of human milk; they resist gastric acids and reach intact the colon, where they selectively influence composition, as well as the function, of the infant gut microbiota. This is obtained by the expansion of beneficial microbial taxa, such as *Bifidobacterium*, capable of positively modulating barrier integrity and regulating systemic inflammation. Such beneficial effects expand beyond intestinal apparatus, as HMOs’ metabolites act as key actors in neurocognitive development, linking early nutrition selective microbial colonization to long-term neurodevelopmental benefits given by breastfeeding [16].

Specifically, in recent years, increasing attention has focused on HMOs as some of the most biologically active components of human milk, with their relevance extending beyond immune protection to neurodevelopmental programming. HMOs, the third most abundant solid fraction in human milk, are resistant to digestion in the upper gastrointestinal tract and reach the colon intact, where they selectively modulate the composition and function of the infant gut microbiota. Through this selective prebiotic action, HMOs promote the expansion of beneficial bacterial taxa, particularly the Bifidobacterium species, supporting intestinal barrier integrity and limiting systemic inflammation. Emerging evidence indicates that HMO-driven microbial metabolism produces bioactive compounds, including short-chain fatty acids (SCFAs) and other neuroactive metabolites, which can influence brain development via the gut–brain axis. In this context, HMOs appear to act as key mediators linking early nutrition, microbial colonization, and neurocognitive development, providing a biological explanation for some of the long-term neurodevelopmental benefits associated with breastfeeding [16].

With this narrative review, we aimed to explore the possible beneficial factors and relationships between microbiota, immunity, and the psycho–neuro–endocrine system of bioactive-supplemented infant formulas.

## 2. From Breast Milk to Bioactive Infant Formulas

Human milk, as previously stated, thanks to its components, represents the biologic and molecular gold standard for neonatal nutrition. Among other factors, the presence of HMOs, along with lactoferrin, cytokines, and live microbes belonging to genera as Bifidobacterium and Lactobacillus transferred from breastmilk [15,17], contributes to generate a favorable ambient to a selected microbiota, improves intestine barrier function, and prevents pathogen adhesion and enhance immune functions [18]. A poorer quality in terms of microbiota composition, and, as a consequence, lesser beneficial effects, can be observed, in neonates using milk formula products [19]. However, human milk, as it could be expected, should not be considered static, both in content and quality. In fact, its component may depend on maternal phenotype and epigenetic, stage of lactation, environmental exposures, and interpersonal variability, making it a dynamic nutrient difficult to reproduce exogenously [15]. Nevertheless, industries produced milk formulas enriched with bioactive components such as prebiotics, probiotics, and postbiotics, in order to mime breastmilk benefits that are not usually elicited by standard infant formulated milk [20]. As a definition we should consider a prebiotic as “a substrate that is selectively utilized by host microorganisms conferring a health benefit” [21], a probiotic as “live microorganisms which when administered in adequate amounts confer a health benefit on the host” [22] and a postbiotic as a “preparation of inanimate microorganisms and/or their components that confers a health benefit on the host” [23]. HMOs, FOS, and GOS fall into the prebiotic category, whereas a wide variety of microorganisms, such as *Lactobacillus*, *Bifidobacterium*, *Saccharomyces*, and *Enterococcus*, represent probiotics. Moreover, the postbiotics category includes: cell-free supernatants substances, taxa-specific proteins solutions capable of modulating inflammatory pathways and controlling microbes proliferation, enzymes, such as bacterial glutathione or peroxidase, peroxide dismutase, adding antioxidant activity, cell wall fragments, including lipoteichoic acid with immunogenic proprieties, bacteriocins, acting as an antimicrobial agent, and SCFAs, which will be further discussed as they are responsible for complex and intricated activities both regarding the host and microbiota itself [24,25]. Other substances, such as α-lactalbumin, the prevalent whey protein in human milk, may not fall exactly in the pre- or post-biotic category, but can exert similar effects [26] and be a relevant and beneficial addition to baby’s milk formula, supporting growth [27] and maintaining good tolerance profiles [28]. This could apply also for long chain polyunsaturated fatty acids (PUFAs), namely docosahexaenoic and arachidonic acids, precursors of, respectively, the essential fatty acid omega-3 and omega-6. Due to their key structural role in neural and retinal tissue [29], and their limited endogenous synthesis in the early life [30], PUFA assumption brings solid outcomes on vision and in long term neuro-development [31]. The European Academy of Paediatrics recommends integration in milk formula of docosahexaenoic and arachidonic acids, in which concentration of the latter should be equal to the former [32]. Consequently, biotics and other molecules, even if not strictly belonging to the biotics category, should be actively considered in the development of infant milk formula, as to emulate breastmilk benefits while providing a safe and rich alternative.

## 3. Molecular Mechanisms of Action

**Figure 1:**
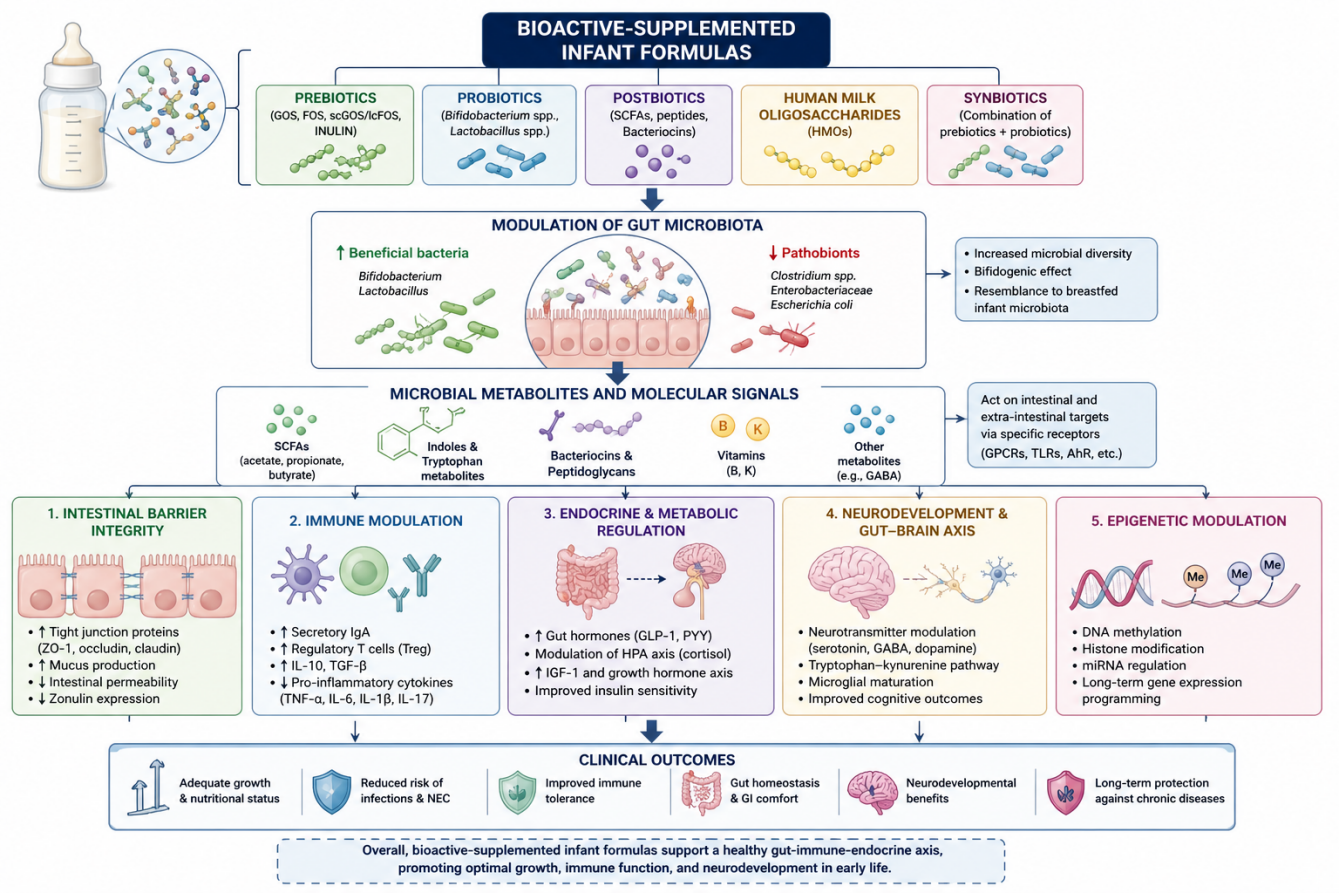
The complex interplay between bioactive compounds, gut microbiota, and host signaling pathways highlights the multidimensional biological effects of enriched infant formulas, extending beyond gastrointestinal modulation toward systemic immune, endocrine, metabolic, and neurodevelopmental regulation.

Nowadays, a new understanding of the relationship between the gut microbiota and the human organism brought the spotlight on modulating health implications attributed to microbiota-related products, namely prebiotics, probiotics, and postbiotics. Said substances are capable, even with different mechanisms, of contributing to intestinal, immune, and metabolic functions, earning themselves a more central role in nutritional and therapeutic alternatives. A schematic overview of the principal molecular pathways linking bioactive-supplemented infant formulas, gut microbiota modulation, and host developmental outcomes is summarized in Figure 1.

Prebiotics, being non-digestible substances, are actively utilized as substrates by intestinal microbiota. In fact, they undergo a fermentation process, mediated by gut bacteria, which produces SCFAs, a wide group of volatile fatty acids, mostly represented by acetate, propionate and butyrate. These substances are produced from the metabolism of amino acids, such as glutamate, succinate, or lysin, through CoA transferase-linked pathways (for example acrylate and succinate), the Wood–Ljungdahl pathway, or saccharides metabolism, via propanediol from fuculose and rhamnulose [33,34]. SCFAs do not represent end-products at all; in fact, they represent active molecules involved in both biological signaling and microbiota crosstalk. SCFAs’ quality and quantity depends on the prebiotic chemical and physical properties (e.g., resistant starch, pectin, β-glucans), and the involvement of different microorganisms [33]. Moreover, SCFA regulates numerous metabolic pathways, acting on specific cellular receptors through G-protein coupled receptors (e.g., GRP41/FFAR3) and influencing endocrine and immune modulation, epithelial integrity, and reduction of the risk of chronic diseases [27,34]. Epigenetics changes are also a part of SCFAs’ metabolic actions; butyrate has been reported to be a potential inhibitor of histone deacetylase and a promoter for Foxo3, resulting in a reduced proliferation in intestinal stem cells [35]. In addition, through cross feeding, prebiotics contributes to maintaining ecological resilience and stability of microbiota community and intestinal homeostasis [27].

Probiotics bring in various health benefits to the host, acting through multiple strain- specific mechanisms [36]. The intestinal barrier represents an example, thanks to probiotic’s capacity to favor secretions of mucins and defensins as well as immune response and thigh junctions’ enhancement [36]. It is reported, moreover, that bacteria strains belonging to the *Lactobacillus* genera (e.g., *L. rhamnosus*, *L. plantarum*) as well as *Bifidobacterium* (e.g., *B. breve*, *B. animalis*) play a central role in stimulating toll-like receptors and IgA production, contributing also to the regulation between pro and anti-inflammatory cytokines [37]. The same substances can control replication of pathogens, through SCFAs and bacteriocins production, maintaining immune tolerance for selected microorganisms. In pre-clinical studies, involving animal colitis models, a restored barrier function and ion transport are reported, as well as reduced epithelial inflammation, which is secondary to probiotics supplementations [38,39]. Probiotics could also be beneficial in neonates facing surgery in the first days of life, even if the scientific literature does not bring definitive results on the topic [40].

Postbiotics and their derived metabolites represent a new approach in microbiota-related therapeutics. Differing from probiotics, postbiotics are capable of exerting beneficial effects independently to microbial intermediaries. Postbiotic-related substances include SCFAs, lipopolysaccharides, muropeptides, secondary bile acids, and peptidoglycans. They, similarly to probiotics, interact with a variety of cell receptors, influencing many processes including immunity, glucose metabolism, epithelial remodeling, and even gut–brain communication (Table 1). The scientific literature, for instance, reports improved insulin sensibility, while other publications flag a possible involvement in metabolic functions, further explained in this review, suggesting a specific context and dose-dependent response profile, worthy of deeper investigations. With no surprise, postbiotic therapeutic potential has been highlighted in complex diseases, including metabolic, neurodegenerative, and oncological ones, allowing clinicians to further choose tailored therapies with additional benefits to the patients [41,42,43].

Synbiotics, as a combination of pro- and prebiotics and being functionally complementary, aim to synergically obtain favorable effects on the host [44,45]. In fact, said complementarity expresses itself by the prebiotic taxa-specific growth support to the probiotic co-administered, as to create a favorable environment allowing probiotic colonization and persistence and therefore enhance the combined pre and probiotics effects, taking also in consideration pre-existing microbiota.

## 4. Effects on Growth and Nutritional Adequacy

Amongst other variables, growth remains one of the key elements evaluated in the matter of infant formula safety and nutritional adequacy, expressed as body weight, length, head circumference, and growth velocity. However, as reported by recent meta-analyses and systematic reviews, supplementation of pre-, post-, or synbiotics does not necessarily result in statistically significant differences in growth when compared to infants using standard infant formulas [46,47]. A meta-analysis, which collected 55 randomized controlled trials and over 8800 infants, did not report any relevant effects on weight, length, or head circumference gain, in the absence of any minor or severe adverse events [48]. A different systematic review, similarly, reports comparable effects of such supplementations on growth in term infants, reporting only a significant softer feces and increased stool frequency in treated patients [47].

However, other studies showed evidence that specific prebiotic FOS and GOS combinations or polydextrose/GOS/lactulose one may be responsible for better weight gain, while others, namely inulin-enriched FOS, appeared to be involved in slight reduction in growth variables [49]. This may rely on composition and duration of said supplements, highlighting even more how biological supplements need to be used with a tailored approach to exert their maximum efficacy. Apart from nutritional equivalence, while comparing infants during their first 12-months of life, synbiotic- and prebiotic-enriched formulas did not show anthropometric differences or safety concerns [50].

Some evidence, however, suggests that specific prebiotic combinations of FOS and GOS or the mixture of polydextrose/GOS/lactulose may promote better weight gain, while others, such as inulin-enriched fructo-oligosaccharides, appear to be associated with a slight reduction in growth indices [49]. This indicates that prebiotic efficacy may depend on the composition and duration of supplementation, highlighting the need for tailored approaches in formula design. Further clinical studies comparing prebiotic- and synbiotic-enriched formulas have reported comparable anthropometric outcomes during the first 12 months of life, confirming their safety and nutritional equivalence [50].

Interestingly, other substances, not considered strictly prebiotic, such as dietary nucleotides, may be responsible for increased anthropometric measures, head circumference, and body weight, in infants consuming a nucleotide-supplemented formula against others using standard formula [51].

Other studies support similar evidences: in specific clinical contexts amino acid-based formulas supplemented with synbiotics have been shown to support normal growth, Burks et al. conducted a prospective, randomized, double-blind controlled trial in which full-term infants with documented cow’s milk allergy received an amino acid-based formula, either alone or supplemented with a specific synbiotic blend containing oligofructose, long-chain inulin, acidic oligosaccharides, and *Bifidobacterium breve* M-16V. Normal growth was observed during the 16-week intervention for both formulas; there was no significant difference between groups in weight, length, or head circumference Z-scores when assessed according to WHO growth standards. This firmly confirms that nutritional adequacy, as well as growth velocity, have not been compromised by the addition of synbiotics in such an at-risk population [52]. The two formulas were well tolerated with reduced allergic symptoms and safety parameters within physical reference ranges during the period of study. From these results, synbiotic-supplemented amino acid-based formulas emerge as a valid and safe alternative to cow’s milk, as they efficiently sustain growth, which is a critical requirement for biological-enriched formulas in the absence of breastfeeding, even if complete replication of the complex human milk is not reached.

## 5. Gut Health and Microbiota Modulation

As previously stated, enriched infant formulas elicit a variety of favorable effects on intestinal health thanks to their action in modulating gastrointestinal function, microbial composition, and production of functional metabolites. Several clinical studies report associations between prebiotic mixtures assumption, such as GOS/FOS (with 9:1 ratio) and improved stool frequency, and reduced fecal pH and softer stool consistency, as active fermentation and good tolerance expression [53,54,55].

Prebiotics and synbiotic assumption reduce potential dysbiosis and its relative markers, promoting selective bacterial taxa growth, mainly *Bifidobacterium* and *Lactobacillus*, and consequently modulating metabolic function of gut microbiota [55,56,57]. Recent metagenomic studies indicate a microbial profile of enriched formula-fed infants closer to the breastfed ones, characterized by a higher prevalence of saccharolytic taxa and reduced presence of *Enterobacteriaceae* [55,56,58]. A recent multi-center double blinded- trial, comparing fecal biomarkers as well as *Bifidobacterium* and *Clostridium* abundance in patients consuming enriched HMOs infant formula versus the ones consuming standard cow milk-based formulas, reported a shift towards a more human-milk-like fecal biomarker profile, in secretory IgA, alpha-1-antitrypsin, and calprotectin, along an improved *Bifidobacterium* proliferation in the former group [58]. Such a microbial shift correlates with an improved intestinal barrier and a minor risk of gastrointestinal disturbances and suggests that enriched infant formulas can have a notable impact on newborns health and should be actively considered in the matter of infant nutrition. A prospective study comparing stool Candida fungal colonization in preterm newborn receiving either *Lactobacillus reuteri*, *Lactobacillus rhamnosus*, or no probiotic supplementation, reported a lower Candida stool colonization, along with fewer gastrointestinal symptoms, days of antibiotic treatment, and less days spent in the neonatal intensive care unit compared to the control group (with generally better outcome in *reuteri*-supplemented patients than *rhamnosus* ones). This supports the proliferation-inhibiting pressure of the probiotic-favored microorganisms, preventing also fungal proliferation [59].

The early-life gut colonization also contributes to long- and short-term host health: a recent metagenomic and metabolomic study indicates how human milk microbiota positively influence infant neurodevelopment. In particular, microbiota-derived metabolites, especially SCFAs, appear to be the central mediator enabling enhanced intestinal integrity as well as developing the gut–brain axis [60]. A similar mechanism of action can be seen in pre, post and probiotics. For instance, upregulation of genes coding for proteins including zonula occludens-1 claudin, in response to exopolysaccharides, is reported to activate the STAT3 pathway and therefore promote thigh junctions’ gene transcription and improve the intestinal barrier [61]. In addition, other microbial-derived metabolites, such as tryptophan, indoles, and derivates, alongside SCFAs derived by pro, pre, and postbiotics, have been linked to better regulation of intestinal inflammation and immune responsiveness, via aryl-hydrocarbon receptor activation, promoting favorable gut microbiota proliferation and, in vivo, a reduction in incidence of necrotizing enterocolitis in neonates [62].

How different feeding strategies, and in particular, supplemented formulas may influence gut metabolomic profile? Regarding this, a recent prospective observational cohort study from Bernardo et al., provided interesting results, comparing for the first time the faecal metabolomic characteristics of late preterm infants fed breast milk, standard formula, or formula supplemented with a postbiotic preparation containing vitamin D3 and fermented fructo-oligosaccharides derived from *Lactobacillus paracasei* [63]. In particular, an overlap between the faecal metabolome of breastfed and formula-fed infants was observed, supporting the idea that modern formulas can at least partially replicate the metabolic imprinting of human milk. Notably, the metabolomic profile of infants receiving postbiotic-enriched formula tended, over time, to cluster more closely with that of breastfed infants than with those receiving standard formula, suggesting that the addition of specific bioactive components may promote a more “breast milk-like” functional adaptation of the gut ecosystem. Notably, breastfed and postbiotic-fed infants shared several metabolites useful for intestinal health: azelaic acid, a marker of healthier inflammatory profiles, was consistently higher in both breast milk and postbiotic groups, while N-acetylglucosamine-6-sulfate, a substrate that supports Bifidobacterium growth and mucosal integrity, was also common to these two groups [63]; reduced glutathione, a key indicator of antioxidant activity, instead, was lower in stools from breastfed and postbiotic-fed infants, reflecting greater biological utilization rather than deficit. To summarize, this study concluded that a closer microbiota environment can be obtained in infants using postbiotic-enriched formula, compared to the ones using standard formula, supporting the role of postbiotics in intestinal homeostasis modulation.

However, in the present day, there is no homogeneity in dosage and strain adopted in therapeutic settings. This results in an added difficulty grade in establishing definitive causal relationships between products used and host well-being, and multi-omics studies are needed to confirm and establish clinical impact of bioactive supplements in infants [56,57,60].

## 6. Microbiota- and Probiotic-Mediated Regulation of Intestinal Stem Cells

The gut microbiota plays an important role in maintaining gastrointestinal homeostasis by influencing epithelial integrity, immunity, and metabolism.

Recent studies have begun to uncover how microbiota-derived metabolites, such as short-chain fatty acids (SCFAs), tryptophan metabolites, and secondary bile acids, interact with stem cells in various segments of the GI tract. These interactions affect stem cell quiescence, proliferation, and differentiation, ultimately affecting tissue integrity and disease susceptibility. Dysbiosis, a disruption of the normal microbial community, has been implicated in the pathogenesis of GI disorders, including inflammatory diseases and cancers, many of which involve alterations in stem cell function.

Stem cells are characterized by the ability to self-renew without losing their developmental potential and to differentiate into multiple specialized cell types. Actively proliferating stem cells, such as Lgr5 + stem cells, may transiently enter a quiescent state under certain conditions. However, their contributions to regeneration following injury remain unclear. Alternatively, unreserved stem cells are defined by their ability to remain quiescent during homeostasis and become activated upon injury or stress to self-renew and differentiate. Therefore, quiescent stem cells can only be classified as reserve stem cells if they have been functionally validated to contribute to tissue regeneration in response to injury [64].

The balance of stem cells is maintained by the niche. The intestinal epithelial stem cell (IESC) niche constitutes a network of cell types expanding well beyond the epithelial layer to help govern the balance between IESC self-renewal and differentiation. The mammalian IESC is composed of epithelial cells including IESCs, Paneth cells, and enteroendocrine cells, as well as stromal, neural, and immune cell types. It is evident that gut microbiota has an important influence on intestinal epithelial physiology and stem-cell function. However, the underlying mechanisms remain poorly understood and are still under active investigation. The development of probiotics or engineered bacteria, as well as molecular strategies represent exciting possibilities for modulating the gut microbiome and the IESC stem cell niche and thereby modify intestinal physiology. Such efforts could, in the long-term, provide benefit to patients with a wide range of gastrointestinal diseases [65].

A recent study has identified specific microbial mechanisms that directly promote epithelial regeneration. This study identifies *Blautia coccoides* as a key commensal bacterium that promotes intestinal epithelial stem cells (ISCs) regeneration by activating HOPX^+^ reserve intestinal stem cells (rISCs) to restore the LGR5^+^ ISC compartment, a process enhanced through metabolic cooperation with indole-3-propionic acid (IPA)-producing bacteria. Inflammatory bowel disease (IBD) is associated with microbial dysbiosis and a consistent depletion of *B. coccoides*, and experimental models demonstrate that BC attenuates colitis severity and enhances mucosal barrier function. *B. coccoides* enhances β-hydroxybutyrate production in intestinal epithelial cells, leading to activation of HOPX^+^ rISCs, which regenerate the LGR5^+^ ISC pool and play a critical role in epithelial repair. These findings support the plasticity and heterogeneity of rISCs populations during injury, highlighting a HOPX^+^-dependent regenerative mechanism. Furthermore, *B. coccoides* generates indole-3-propionic acid and synergizes with other bacteria to produce IPA, emphasizing the importance of microbial co-metabolism in shaping host-active metabolites. This study demonstrates the therapeutic potential of targeting microbial metabolic networks to stimulate endogenous repair mechanisms. In conclusion, *B. coccoides* promotes intestinal epithelial regeneration through a β-hydroxybutyrate–HOPX^+^ rISC axis, amplified by metabolic interactions, highlighting its relevance for epithelial homeostasis and repair in IBD and related conditions [66]. In the continuous regeneration of intestinal epithelium of intestinal stem cells, *Lactobacillus reuteri* D8 has been shown to protect intestinal mucosa integrity both in organoid models and in vivo. A study found that only live *L. reuteri* D8 was effective in protecting the morphology of intestinal organoids and normal proliferation of epithelial stained with 5-ethynyl-2′-deoxyuridine under tumor necrosis factor-α treatment, which was also further verified in mice experiments. *L. reuteri* protects intestinal barrier and activates intestinal epithelial proliferation, which sheds light on treatment approaches for intestinal inflammation based on ISCs with Lactobacillus probiotics and daily probiotic consumption in healthy foods [67]. Another study utilized in vivo broilers plus an ex vivo organoids model to thoroughly examine the effectiveness of *L. reuteri* in protecting the integrity of the intestinal mucosa during lipopolysaccharide-induced (LPS-induced) enteritis in broilers. This research revealed that *L. reuteri* promoted the expansion of ISCs and intestinal epithelial cell renewal by regulating the Wnt/β-catenin signaling pathway, thereby maintaining the integrity of the intestinal mucosal barrier. This finding provided theoretical support for lactobacillus as a probiotic additive in livestock feed to improve intestinal inflammation and treat intestinal diseases [68].

Mesenchymal stem cells are influenced by biotic factors too: a recent study, conducted by Goudarzi F. and colleagues, highlights the effect of *Lactobacillus delbrueckii* on human adipose-derived mesenchymal stem cells, suggesting *L. delbrueckii* subsp. lactis KUMS-Y33 can redirect the differentiation potential of mesenchymal stem cells away from adipogenesis and promotes osteogenesis in human adipose-derived mesenchymal stem cells, suggesting a positive role in the prevention and treatment of osteoporosis and opening a new aspect for future in vivo study [69].

## 7. Immune Development and Clinical Outcomes

New scientific evidence reports positive immune modulation and development associated with better clinical outcomes regarding infectious diseases in infants consuming supplemented milk formulas with probiotics, prebiotics, or postbiotics during early life. In fact, in randomized controlled trials, probiotic-supplemented formulas, in particular, the ones containing *Bifidobacterium infantis*, *Bifidobacterium bifidum*, and *Lactobacillus helveticus*, showed sustained fecal secretory IgA levels, comparable to breastfed infants. This does support mucosal immune maturation and improve gut barrier function [70,71]. Incidence, duration of respiratory tract infection, and its morbidities can also be influenced positively by enriched infant formula, suggesting an immune system improved activity and reactiveness, especially during the first days of life [72]. In addition, as shown by a systematic review with network meta-analysis, use of said formulations appears to be safe, tolerated, and potentially capable of lowering antibiotics use and incidence of febrile episodes [73]. Probiotic acts on the immunoregulatory side too: in particular, some clinical studies report that propionate promotes the development or regulatory T-cells, and, simultaneously, reducing levels of TNF-α, rising IL-10 production an enhancing dendric cell survival and maturation [25]. These findings correlate to the early-life microbial colonization and proliferation through biotic supplementations, which manifests itself via the more robust mucosal immune defense and the consequent reduced risk of pathogens infections, although the magnitude of said benefits may vary depending on strain, dosage, and duration of administrations. In addition, likely through microbes’ proliferation control and preservation of mesenteric perfusion, HMOs and a specific isomer of disialyllacto-N-tetraose, in neonatal necrotizing enterocolitis rat models, improved survival rates, even if GOS did not sort the same effects [74]; similar effects have been observed with 2′-fucosyllactose′-induced expression of endothelial nitric oxide synthase [75]. Additionally, integration of bovine lactoferrin, synergically with *L. rhamnosus* GG probiotics can reduce necrotizing enterocolitis incidence very-low-birth-weight neonates [26]. This underlines the notable impact of human milk microbiota-favoring substances on newborns, especially in pathological scenarios such as necrotizing enterocolitis [26,75,76,77,78], and the metabolite-specific interactions that postbiotics can express. However, these findings should be further studied through shared and standardized criteria, as to obtain evidence on a large population, since contradictory evidence on lactoferrin actually scale down the benefits found by other studies [79]. Immunomodulatory postbiotics effects are involved in autoimmune regulations too. In fact, links between inflammatory bowel syndromes, and postbiotic-induced changes in epithelial barrier function, as well as antioxidant activity and butyrate-producing bacteria proliferation, are widely described in the scientific literature [71]. Studies involving long-term clinical follow-up, integrated with immunological biomarkers, may unravel more on the impact of biotic-supplemented formulas and the induction of immune programming and tolerance.

Regarding clinical outcomes, the analysis conducted by Cool and Vandenplas links several studies demonstrating a significant reduction in total infections, particularly respiratory and gastrointestinal infections, during the first months of life [80]. Furthermore, research such as that of Arslanoglu et al. on HMOs demonstrates a reduction in infections of up to 46% and a favorable immune modulation similar to breastfeeding [81].

## 8. Obesity and the Gut–Brain Axis

The gut microbiota plays a major role in human health and disease—a dysbiotic composition evident in obese and aged individuals. The bidirectional communication system between the gut and the central nervous system, known as the gut–brain axis, may link obesity to unhealthy aging.

For this reason, a growing interest has developed into the role of microbiome-based interventions, including probiotics, prebiotics, and synbiotics, in modulating metabolic outcomes such as obesity via the gut–brain axis, a bidirectional system through which the gut microbiota influences physiology, including appetite regulation, energy balance, and metabolic homeostasis [82]. As highlighted in the literature, gut microbiota plays a central role in nutrient metabolism, immunomodulation, and maintenance of intestinal barrier integrity, and is strongly influenced by dietary habits. Alterations to this microbial ecosystem, known as dysbiosis, are associated with obesity and are characterized by reduced bacterial diversity, shifts in microbial composition, and functional impairments such as altered production of SCFAs and other metabolites [83]. These changes can promote increased gut permeability, allowing bacterial components such as lipopolysaccharides (LPS) to enter systemic circulation and cause chronic low-grade inflammation, which contributes to metabolic dysregulation and adipocyte dysfunction [84]. In this context pre-, post- and synbiotics can restore microbial homeostasis. Evidence suggests that they can reduce inflammation, improve lipid and glucose metabolism, and positively influence energy homeostasis, although their effects are often strain-specific and influenced by external factors such as diet, geography, and lifestyle [85]. This topic is particularly relevant in early life, when the gut microbiota is still developing and is highly susceptible to environmental influences, including infant feeding practices. The gut microbiota changes throughout the lifespan and reaches relative stability in adulthood; however, early-life alterations may have long-lasting effects on metabolic health [86]. Notably, microbiota alterations may impair gut eubiosis and influence the development of the gut–brain axis, potentially predisposing individuals to dysregulated appetite control, increased energy harvest, and a higher risk of childhood obesity [87]. Established that diet is a major determinant of microbiota composition, early nutrition enriched with prebiotics, probiotics, or synbiotics are being explored as strategies to shape a healthier microbial profile from infancy [88].

Moreover, a study, from Lee K and colleagues pays particular attention to the browning of white adipose tissue (WAT). This is a process where WAT develops characteristics of brown adipose tissue (BAT), including thermogenic capability. This process has emerged as a promising therapeutic target, as it induces loss of body mass and improves blood glucose control. In this context, probiotics, particularly *Lactobacillus* strains, have attracted attention due to their worthwhile effects on human health, including anti-inflammatory, anti-diabetic, and anti-hypercholesterolemic properties. *Lactobacillus* strains are known to exert inhibitory effects on obesity through the reduction of fat accumulation and may enhance heat generation through the browning of WAT. Experimental evidence demonstrates that supplementation with a mixture of *Lactobacillus curvatus* HY7601 and *Lactobacillus plantarum* KY1032 significantly ameliorates high-fat diet-induced increases in body weight and adipose tissue mass, while decreasing lipid droplet size and improving metabolic parameters such as fasting blood glucose levels. These effects are associated with increased thermogenesis, as indicated by elevated core temperature and upregulation of key thermogenic markers, including UCP1, PGC1α, and SIRT1, which promote fatty acid oxidation and the differentiation of white adipocytes into beige adipocytes. This probiotic mixture improves lipid metabolism by reducing serum triglycerides, total cholesterol, and LDL-cholesterol, while increasing HDL-cholesterol and adiponectin levels. It also enhances cholesterol disposal by increasing fecal excretion of cholesterol and bile acids and upregulating genes involved in cholesterol transport and metabolism, such as *Abcg5*, *Abcg8*, *Lxra*, and *Lxrb*. These findings suggest that probiotics may promote energy expenditure through fatty acid β-oxidation and thermogenesis, while simultaneously regulating cholesterol homeostasis. Overall, this evidence supports the concept that specific probiotic strains can modulate energy metabolism, adipose tissue function, and lipid balance, highlighting their potential as microbiome-based therapeutic strategies for obesity [89].

## 9. Psycho–Neuro–Immuno–Endocrinological Microbiota Involvement

Data on endocrine-related effects on enriched baby milk formula are scarce. It is reported an improved sleep–wake pattern in infants receiving a prebiotic blend with cow milk-based formula, linked to cortisol awakening saliva levels [90]. However, evidence on singular microbiota-associated substances can provide some insights and a potential hypothesis that could relate to an in vivo setting: HMOs as well as GOS and FOS fermentation products, and SCFAs, among other metabolites, are capable to stimulate, through L-cell and entero-endocrine cells, GLP-1 and PYY release, as well as, interestingly, increased entero-endocrine cells numbers [91], which could have a role into regulating appetite and glucose tolerance since the first days of life. Additional insights can be drawn from animal-model experiments with possible correlations valid for humans too; in fact, in a series of experiments on germ-free mice characterized by microbiota absence, researchers found slower body growth, worse bone formation and length when compared to wild-type ones. Interestingly, this could be reversed after a long-term colonization of Lactobacillus plantarum [92] suggesting a possible involvement of the GH/IGF-1 axis, even if no clear pathway has been yet described [93]. Another curious finding regards the hypothalamus–pituitary–adrenal axis, which can also be affected by microbiota. Similarly, in the germ-free mice model, plasma ACTH and corticosterone levels has been found higher when compared to gnotobiotic mice and this reverted after *Bifidobacterium infantis* administration [93]. Accordingly, a recent systematic review and metanalysis reported lowered cortisol levels after supplementation with probiotics, though a higher evidence level is needed, due to variability in used probiotic strain and population sample heterogeneity [94]. Furthermore, it is reported that microbiota circadian variability correlates with cortisol levels, involving sleep–wake cycle, disrupted in animals with a microbiota depleted through antibiotic treatment [95] suggesting an even deeper connection between gut microbes and human organisms.

With no surprise this could represent a fraction of a more complex psycho–neuro–endocrine–immunologic implications elicited by brain and gut cross-communication. In fact, many studies have been published regarding microbiota-produced substances, such as kynurenine, tryptophan, and serotonin, and their role on modulating neuronal development and immune regulation [96,97,98] fully capable to program neuronal organization and possibly impacting a variety of psychiatric/neurological diseases [90] as well as imposing positive effects on development of motor skills, social abilities, better scores on checklist and behavioral scales associated with Bifidobacterium and Bacterioides taxa, and worse adaptive and communication skills for Clostridium and some Lachnospiraceae.

**Figure 2:**
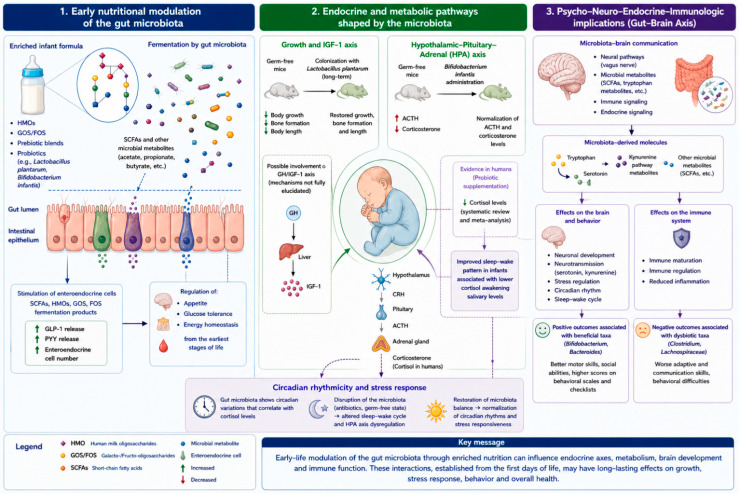
Effects of microbiota and its derived molecules in the early-life nutrition and long-term consequences in metabolic, endocrine, neuro-developmental, and immune system.

A scheme of the complex interaction between microbiota and its derived molecules, with neurological, immune and endocrine system can be consulted in Figure 2.

## 10. Safety and Tolerability

Current evidence indicates that the supplementation of infant formulas with probiotics, prebiotics, synbiotics, and postbiotics generally exhibits a favorable safety profile and good gastrointestinal tolerability. Multiple randomized clinical trials and systematic reviews have demonstrated that such supplementation does not negatively affect growth, body composition, or key clinical parameters compared with standard formulas [73,99,100,101]. Supplementation of GOS and FOS, as well as probiotics consumption containing *Bifidobacterium breve* and *Lactobacillus reuteri* has been associated with incidence reduction of infantile colic, constipation, and diarrhea with an overall improved and regular intestinal function [54,73,100]. Additionally, several studies report a reduced feeding intolerance, and risk of necrotizing enterocolitis in the preterm population [26,78,102], exposing the preventive role of a regulated microbiota induced by probiotic consumption.

However, along with other products used for human consumption, environmental contaminants and endocrine-disrupting substances, originating form packaging and industrial processes, have been an element of concern. Estrogen-like substances, phthalates, and bisphenols have been found in certain infant formula batches and human milk itself, rising the concern about monitoring and integrating toxicological assessment for such products [102,103]. Some of these substances, used in manufacturing processes, can influence shelf life and stability of bioactive products and therefore could have a role in preservation of biological proprieties and microbial viability [104].

Safety assessments conducted by EFSA (2014) and ESPGHAN (2011) concluded that there is no evidence of safety concerns or clinically significant benefits related to the addition of probiotics to infant formula [99]. More recent analyses, including 17 systematic reviews and meta-analyses, confirm the absence of major safety concerns in both healthy and high-risk infants, while emphasizing that many studies exhibit methodological limitations and are not specifically designed to monitor adverse events systematically. 

Current evidence supports a cautious and well-monitored use of biotics in infant formulas, as they appear to be well tolerated and safe in most cases. However, methodological heterogeneity and the lack of long-term longitudinal studies warrant further investigation to elucidate potential endocrine and immunological effects, as well as to strengthen the regulatory and manufacturing foundations of these innovative nutritional approaches.

## 11. Research Gaps and Future Directions 

Research about relationships between human milk, enriched formulas, and microbiomes flourished in the recent years and has advanced considerably; however, knowledge gaps persist, limiting the clinical potentialities of the actual findings. Newer “omics” technologies, including proteomics, metabolomics, lipidomics, and glycomics contributed to revealing the biochemical complexities of human milk and its multiple links between maternal and infant health implications [105].

Nevertheless, it should be noted that most of the currently available evidence has been obtained from heterogeneous studies, with different methodologies, population, duration, and outcomes measures. Said diversity makes drawing robust and objective conclusions difficult, in the matter of probiotic, prebiotic, or postbiotic impact on infant health, immunity, and growth [46,105]. From this perspective, enabling the scientific community to provide complete, stratified, and standardized evidence, including and integrating multi-omics, endocrine, and immunological variables might be a key strategy to deeply explore co-influence of early nutrition and microbiota and its objective effects on infant population and their development [105,106].

Pediatric nutrition, especially when infant and preterm newborns are taken into account, should be considered a cornerstone in long-term well-being and part of precision and personalized medicine. Integrating microbiome and -omics data could enable health-workers and caregivers to develop personalized strategies tailored to the individual biological profiles of the newborn and the mother. Maternal diet, feeding practices, and quality and timing of food introduction, as recent studies suggests, can modulate significantly the microbiome, and therefore reflect on immunity and the newborn development [46,106,107]. Advances in the supplementation of infant formulas represent a valid opportunity to mimic human milk benefits, through their bioactive components [107].

A relatively novel and emerging frontier in microbiota-related research includes endocrine and immune systems; during breastfeeding, many hormones, such as IGF-1 and leptin are transferred to the newborn, with implications on energy metabolism, growth, bone mineralization, and neurological and immunological development [106]. A deep knowledge of the interplay between regulatory hormones, gut–brain axis, and gut microbiome could bring in new pathways, biomarkers, or novel insights on nutritional alternatives with remarkable roles on next generation artificial milk [106,107,108].

Such wide and intricated influences of microbiota in human biological and biochemical physiology justifies the studied outcomes, used substances, population analyzed, and chosen study protocols fragmentation in the recent studies on the matter. As previously stated, this undermines and hampers cross-study comparisons analysis and interferes with the development of consensus and international guidelines. Evidence-based formulation of infant-destined products needs validated biological endpoints, safety measures, and standardized protocols [105,108]. We hope that integrating precision nutrition, metabolomics, and microbiota may clarify the intricated interactions among the maternal/artificial diet and the infant well-being, but also provide solid evidence on the infant psycho–neuro–immuno–endocrinological axis implications that easily extend on the whole newborn organism [107].

However, significant progress is being made, while at the same time attempting to overcome the ethical limitations of experimental studies in newborns, using intestinal organoid systems, which represent a highly reliable model for studying the effects of prebiotics and probiotics on intestinal stem-cell function, epithelial differentiation, and intestinal–endocrine signaling in early life. In particular, the use of intestinal organoid cultures is proving to be a valuable tool for studying the effects of prebiotics and postbiotics on intestinal cell growth and differentiation in early life [109].

Another interesting study field regards interpersonal microbial transmission. Recent studies show that microbial transmission between individuals occurs at the single-strain level, with high specificity and persistence capacity over time, significantly influencing microbiota composition since the first months of life. Microbiota transmission, between relatives, siblings and other children heavily modulates the composition of the newborn, and the older children also, creating a sort of shared microbiota [110,111]. This adds to the importance of the microbial seeding happening throughout vaginal birth, without downscaling it, manifesting the high transmissibility of bacterial strains.

## 12. Conclusions

Nutrition in the early stages of life, the developing gut microbiota, and the immune, metabolic, and neuroendocrine systems in newborns communicate and interact with each other to create a balance that is crucial in determining long-term health. Clearly, breast milk remains the gold standard for feeding newborns, as it is able to provide nutrients, immune mediators, hormones, and complex oligosaccharides that can influence early microbial colonization. However, breastfeeding is sometimes not possible, so a scientifically based strategy was needed to partially reproduce the fundamental functional aspects of breast milk: formula milk enriched with prebiotics, probiotics, postbiotics, or synbiotics. Current clinical evidence, as demonstrated by our review, has shown that these formulas supplemented with bioactive substances are nutritionally adequate, safe and well tolerated, promoting normal growth even in premature infants. It is precisely at the gastrointestinal level that these enriched milks demonstrate the most evident effects: in fact, they modulate stool characteristics, promote a bifidogenic microbial profile and improve microbial metabolic activity, often modifying the intestinal ecosystem towards models more similar to those of breastfed infants. In addition to its already-known benefits for gut health, recent studies suggest potential benefits for mucosal immunity and the risk of early infections, although these effects appear to depend largely on the strain, dose, and formulation. Furthermore, enriched formulas could have a positive effect on the endocrine and metabolic systems and on the psychoneuroimmunological axis; this effect has a biological explanation, although the mechanisms are still being explored and require confirmation in well-designed longitudinal studies in humans. Results from animal models and preliminary human studies have indicated possible influences on enteroendocrine signaling, the GH/IGF-1 axis, stress responsiveness, and gut–brain communication mediated by microbial metabolites such as short-chain fatty acids and tryptophan-derived compounds. Despite significant progress, major challenges remain. Methodological heterogeneity, limited follow-up duration, lack of standardized biomarkers, and variability in bioactive components hinder the translation of mechanistic knowledge into definitive clinical recommendations. Additionally, issues relating to production processes, bioactive stability and exposure to environmental contaminants require constant regulatory vigilance. In conclusion, formulas enriched with bioactive substances come close to reproducing the biological complexity of breast milk, providing a valuable alternative when breastfeeding is not possible. Future research integrating multi-omic approaches, longitudinal endocrine and immune profiles, and precision nutrition principles will be essential to refine formulae and better support optimal growth, immune competence, metabolic resilience, and neurological development throughout life.

## Figures and Tables

**Table 1:** Principal mechanisms of action of biotics and bioactive molecules.

Mechanism Pathway	Biological Impact and Functional Outcome
SCFA Production	Fermentation of prebiotics produces short-chain fatty acids (acetate, propionate, butyrate) in biological signaling and microbiota crosstalk
Cellular Signaling	SCFAs act on specific G-protein coupled receptors (e.g., GRP41/FFAR3) to influence endocrine and immune modulation [27,28].
Epigenetic Modulation	Butyrate acts as a histone deacetylase inhibitor and Foxo3 promoter, regulating the proliferation of intestinal stem cells [29].
Barrier Enhancement	Probiotics stimulate mucin and defensin secretion and enhance tight junctions to maintain intestinal barrier integrity [30,31,32,33].
Postbiotic Signaling	Postbiotic metabolites interact with cell receptors to influence glucose metabolism, epithelial remodeling, and gut–brain communication [35,36,37].
Synbiotic Synergy	Complementary action where prebiotics provide strain-specific growth support for co-administered probiotics [38,39].

## Data Availability

No new data were created or analyzed in this study. Data sharing is not applicable to this article.

## Abbreviations

The following abbreviations are used in this manuscript:

ACTH	Adrenocorticotropic hormone
EFSA	European Food Safety Authority
ESPGHAN	European Society for Paediatric Gastroenterology, Hepatology and Nutrition
FFAR3	Free fatty acid receptor 3
FOS	Fructo-oligosaccharides
GH	Growth hormone
GLP-1	Glucagon-like peptide-1
GOS	Galacto-oligosaccharides
GRP41	G protein-coupled receptor 41
HMOs	Human milk oligosaccharides
HMO	Human milk oligosaccharide
HPA axis	Hypothalamic–pituitary–adrenal axis
IgA	Immunoglobulin A
IGF-1	Insulin-like growth factor 1
IL	Interleukin
ISAPP	International Scientific Association for Probiotics and Prebiotics
NEC	Necrotizing enterocolitis
NICU	Neonatal intensive care unit
PYY	Peptide YY
SCFAs	Short-chain fatty acids
STAT3	Signal transducer and activator of transcription 3
TNF-α	Tumor necrosis factor alpha
WHO	World Health Organization
